# Renal lithiasis and nutrition

**DOI:** 10.1186/1475-2891-5-23

**Published:** 2006-09-06

**Authors:** Felix Grases, Antonia Costa-Bauza, Rafel M Prieto

**Affiliations:** 1 Laboratory of Renal Lithiasis Research, Universitary Institute of Health Sciences Research (IUNICS), University of Balearic Islands, E-07122 Palma de Mallorca, Spain

## Abstract

Renal lithiasis is a multifactorial disease. An important number of etiologic factors can be adequately modified trough diet, since it must be considered that the urine composition is directly related to diet. In fact, the change of inappropriate habitual diet patterns should be the main measure to prevent kidney stones. In this paper, the relation between different dietary factors (liquid intake, pH, calcium, phosphate, oxalate, citrate, phytate, urate and vitamins) and each type of renal stone (calcium oxalate monohydrate papillary, calcium oxalate monohydrate unattached, calcium oxalate dihydrate, calcium oxalate dihydrate/hydroxyapatite, hydroxyapatite, struvite infectious, brushite, uric acid, calcium oxalate/uric acid and cystine) is discussed.

## Introduction

Renal lithiasis can be defined as the consequence of an alteration of the normal crystallization conditions of urine in the urinary tract. In a healthy individual, during the residence time of urine in the urinary tract, crystals either do not form or are so small they are eliminated uneventfully (asymptomatic crystalluria). When normal urine crystallization conditions become altered, however, the rate of crystal nucleation and growth may become such that the crystals cannot be easily eliminated due to their size. In some cases, altered urinary conditions affecting crystallization are related to specific underlying disorders such as hyperparathyroidism, which is associated with hypercalciuria [1]; tubular acidosis, which is associated with hypercalciuria and hypocitraturia [2]; and some genetic alterations, which are associated with hyperoxaluria [3], hypercystinuria [4] and hypercalciuria [5]. However, in many cases it is not possible to clearly identify the underlying disorder. Indeed, in nearly all renal calculi cases, crystal formation is attributable to a combination of diverse factors that may or may not be associated with an underlying disorder. These factors can be classified into two main groups: urine composition factors and renal morphoanatomy factors.

Urine composition factors are important in crystal formation as urine is a metastable liquid containing several coexisting substances that can crystallize to generate renal calculi. These substances are present at supersaturated levels (the system contains higher amounts of solute than that corresponding to the solubility), meaning the urine is in an unstable state, and a stable urine state will eventuate through crystallization of the excess solute. The ease of crystallization depends on the degree of supersaturation, the presence of preformed particles (so-called heterogeneous nucleants that act as promoter substances) and the level of crystallization inhibitors. These latter substances inhibit crystal nucleation and/or growth.

There are two main renal morphoanatomy factors that can affect crystal formation. The first of these is the presence of cavities (formed by renal calices) with low urodynamic efficacy that retain urine for long periods. The second is an altered epithelium covering the renal papillae, which can arise from events such as damage to the anti-adherent glycosaminoglycan layer that covers the uroepithelium, necrosis, or the presence of subepithelial calcifications.

The development of renal calculi usually involves both urine composition and renal morphoanatomy factors. Thus, not all people with hypercalciuria, hypocitraturia or hyperuricuria, for example, will develop renal calculi. The effect of a factor on renal lithiasis depends on the nature and magnitude of other factors when a particular renal stone is generated. For example, while some alterations to the uroepithelium alone may not be serious enough to cause stone formation, when combined with other factors stone formation might occur. Thus, precise knowledge of the factors involved in the development of a given renal calculus is vital, and adequate study of the calculus structure and composition allows identification of an important number of possible etiologic factors related to its formation. Many such factors can be adequately modified through diet, as this has a significant effect on urine composition. Indeed, altering inappropriate habitual dietary patterns should be the main measure for preventing kidney stones. There are thorough recent literature reviews on nutrition and renal lithiasis that discuss numerous dietary factors and their effects on urine composition and stone formation [6,7].

The present paper discusses the relationship between different dietary factors and each type of renal stone [8], using the classification indicated in Table 1. A key aspect in preventing all kinds of kidney stone is suitable liquid intake, which affects both the urine concentration and the frequency with which solid microparticles are expelled from the urinary system. A study of idiopathic calcium oxalate stone formation demonstrated that the initial therapy for the prevention of any type of kidney stone recurrence is increased fluid intake to ensure a urine volume of at least 2 liters per day [9].

**Table 1:** Renal stone classification

Renal stone classification [8]	Percentage (%)
• Calcium oxalate monohydrate papillary	13
• Calcium oxalate monohydrate unattached (formed in renal cavities)	16
• Calcium oxalate dihydrate	34
• Calcium oxalate dihydrate/hydroxyapatite mixed	11
• Hydroxyapatite	7
• Struvite infectious	4
• Brushite	1
• Uric acid	8
• Calcium oxalate/uric acid mixed	3
• Cystine	1

### Type of renal stone and dietary advice

#### Calcium oxalate monohydrate papillary calculi

Formation of calculi of this type implies the existence of an altered (damaged or partially injured) papillary epithelium. This can be a consequence of cytotoxic substances that also can induce subepithelial calcifications [10,11]. Moreover, renal calculi of this type are frequently associated with a deficiency in the levels of crystallization inhibitors and hyperoxaluria. Citrate and phytate are the only crystallization inhibitors that can be taken via the diet or as a pharmaceutical.

Citrate consumption (through citrate-containing foods or as potassium or sodium salt drugs) causes an increase in urinary pH, which results in increased citrate excretion [12]. In this case, the urinary pH must be controlled to avoid calcium phosphate crystallization. Citrate decreases calcium salt supersaturation due to its capacity to complex calcium ions, and also has crystallization inhibitor activity [13].

Phytate, mainly present in whole cereals and legumes (see Table 3), can inhibit calcium salt crystallization [14-17], and individuals prone to calcium oxalate stone formation were found to have lower urinary phytate excretion compared to healthy subjects [18]. Data from two large observational epidemiological studies revealed an inverse association between phytate intake and the risk of stone formation in women [19].

**Table 2:** Relationship between urinary lithogen factors, types of renal calculi and dietary recommendations

Urinary Lithogen Factor	Values of potentially lithogenic urinary biochemical parameters	Type of renal calculi	Dietary recommendations
pH	< 5.5	COM u UA COM/UA CYS	Decrease habitual consumption of: • Animal protein Increase habitual consumption of: • Citrus juices • Soft-drinks • Citric acid rich beverages

pH	> 6.0	COM p COM u COD HAP COD/HAP BRU	Decrease habitual consumption of: • Vegetarian diet • Citrus juices • Soft-drinks • Citric acid rich beverages

Calcium	>170 mg/L female: >250 mg/24 h male: >300 mg/24 h	COD HAP COD/HAP	Increase habitual consumption of: • Water intake (> 2 l/day) Decrease habitual consumption of: • Sodium • Animal protein Control: • Vitamin D consumption • Calcium supplements

Oxalate	> 40 mg/24 h	COM p COM u	Decrease habitual consumption of: • Oxalate rich foods (see Table 4) • Ascorbic acid rich foods (vitamin C intake greater than 2 g/day)

Citrate	< 350 mg/24 h	COM p COM u COD HAP COD/HAP	Increase habitual consumption of: • Citrate rich foods • Citric acid rich beverages

Phytate	< 1 mg/24 h	COM p COM u COD BRU	Increase habitual consumption of: • Phytate rich foods (see Table 3)

Urate	> 650 mg/ml female: > 600 mg/24 h male: > 800 mg/24 h	UA COM/UA	Decrease habitual consumption of: • Purine rich foods (see Table 5) • Alcoholic drinks

COM p: Calcium oxalate monohydrate papillary

COM u: Calcium oxalate monohydrate unattached (formed in renal cavities)

COD: Calcium oxalate dihydrate

COD/HAP: Calcium oxalate dihydrate/hydroxyapatite mixed

HAP: Hydroxyapatite

STR: Struvite infectious

BRU: Brushite

UA: Uric acid

COM/UA: Calcium oxalate/uric acid mixed

**Table 3:** Main phytate-rich foods

Phytate Rich Foods [45]
• Cereal germ: i.e. corn germ
• Cereal bran: i.e. wheat cereal (100% bran)
• Whole cereals: i.e. wild rice
• Beans: i.e. whole bean, bean flours, bean protein products such as tofu
• Nuts: i.e. brazil nuts

Oxalate-rich foods may be a risk factor for formation of calcium oxalate monohydrate papillary calculi (see Table 4). A dietary oxalate excess is related to formation of calcium oxalate monohydrate (COM) calculi and can mainly be related to an excess consumption of soybean seeds, nuts, spinach, chocolate and green tea [20]. However, there are high intra-individual variations to the contribution of ingested oxalate to urinary oxalate excretion. The urinary exogenic oxalate can account from approximately 10% [21] to over 50%, in particular in the presence of oxalate hyperabsorption or in presence of an adverse calcium/oxalate ratio in the gut [22]. Moreover, it is also necessary to consider ascorbic acid (vitamin C), which is an oxalate precursor. A study involving 186 calcium oxalate stone formers with and without hyperoxaluria demonstrated an inverse association between urinary oxalate excretion and dietary calcium intake, and a positive relationship with dietary ascorbate [7]. A human study involving stone formers and non-stone formers who ingested 2000 mg/day ascorbic acid demonstrated increased urinary oxalate excretion in 40% of all participants (both stone formers and non-stone formers) [23].

**Table 4:** Main oxalate-rich foods

Oxalate Rich Foods [20]
• Spinach
• Rhubarb
• Purslane
• Parsley
• Lambsquarters
• Chives
• Beet leaves
• Amaranth
• Green tea
• Chocolate

Unfortunately, a simple table is not adequate for comparing oxalate-rich foods since: (a) the relative amounts of soluble and insoluble oxalate affect oxalate absorption, and simple percentage lists make no such distinctions, (b) the oxalic acid content can vary substantially depending on the environment in which the food source was grown, and (c) the amount of oxalate ingested is affected by the methods of food preparation and cooking, and the serving size.

### Calcium oxalate monohydrate unattached calculi (formed in renal cavities)

The main etiologic factors for these calculi are the existence of cavities with low urodynamic efficacy, the presence of heterogeneous nucleants mainly constituted by organic matter, hydroxyapatite crystals at a urinary pH ≥ 6.0 or uric acid crystals at a urinary pH ≤ 5.5, and a deficiency in the crystallization inhibitors citrate and phytate [24]. Hyperoxaluria is also a factor frequently associated with this type of renal calculi [25]. Dietary habits associated with high oxaluria and crystallization inhibitor deficiency are discussed above and summarized in Table 2.

Urinary pH is an important variable that can be strongly affected by diet. A diet rich in animal protein is associated with high uric acid urinary excretion and a low urinary pH [26,27]. Uric acid solubility decreases dramatically at a urinary pH lower that 5.5, leading to uric acid crystal formation that can act as a heterogeneous nucleant for calcium oxalate crystals [28]. For people eating a vegetarian diet, the consumption of citrate-rich products (foods and soft-drinks) and carbonated beverages notably increases the urinary pH [26,27,29]. In this case, it is important to consider that calcium phosphate solubility abruptly decreases at pH values above 6.0, causing formation of calcium phosphate crystals that can act as heterogeneous nucleants for calcium oxalate crystals [30]. Indeed, hydroxyapatite and uric acid are very frequently found at the core of renal calculi of this type [8]. The dietary recommendations for urinary pH control are summarized in Table 2.

### Calcium oxalate dihydrate calculi

These calculi generally develop in cavities of low urodynamic efficacy and are associated with hypercalciuria, crystallization inhibitor (citrate and phytate) deficiency and in some cases with urinary pH values greater than 6.0 [8]. Dietary aspects related to crystallization inhibitors and urinary pH > 6.0 are discussed above and summarized in Table 2. Recent studies demonstrate that an increase in urinary calcium excretion is mainly associated with a high consumption of sodium and animal protein rather than consumption of dietary calcium [17,31]. Consequently, a decrease in the consumption of sodium and animal protein is recommended for reducing urinary calcium levels. Also excess consumption of Vitamin D with calcium supplements can induce excessive urinary calcium excretion [30,32]. However, decreasing urinary calcium concentration through restriction of dietary sodium and animal protein is often not completely effective and hypercalciuria persists. In such cases, the type of hypercalciuria should be determined and drug treatment may be necessary [33,34].

### Calcium oxalate dihydrate/hydroxyapatite mixed calculi

These calculi are associated with hypercalciuria, a urinary pH > 6.0, and hypocitraturia [8]. The existence of cavities with low urodynamic efficacy favours the formation of these calculi. Dietary aspects related to urinary pH and calcium and citrate excretion are discussed above and summarized in Table 2. In several cases, hypercalciuria detected in these stone formers is associated with hyperparathyroidism that must be treated [1,35].

### Hydroxyapatite calculi

The etiologic factors related to these calculi are a urinary pH > 6.0, hypocitraturia, hypercalciuria, hyperphosphaturia and hypomagnesiuria. Formation of these calculi is also favoured by the existence of cavities with low urodynamic efficacy. Dietary aspects related to urinary pH and calcium and citrate excretion are discussed above and summarized in Table 2. The urinary phosphate is related with phosphate intake [36], but as in the case of oxalate, dietary phosphate restriction increases calcium urinary excretion [37]. For this reason, the decrease in phosphate intake (restriction of milk, cheese, fish, sausages, soft drinks with phosphoric acid) should go in with a calcium intake reduction [38] and apply only in hyperphosphaturic states. The formation of these calculi is occasionally associated with the existence of renal tubular acidosis that requires specific pharmacological treatment [39].

### Struvite infectious calculi

Formation of these calculi is due to urinary infection, and thus treatment involves pharmacological (antibiotic) intervention [40]. To prevent recurrent infections, it is recommended that urinary pH values be maintained below 6.0. The dietary influence on urinary pH is discussed above and summarized in Table 2.

### Brushite calculi

The etiologic factors for these calculi are a urinary pH ≥ 6.0, crystallization inhibitor (phytate and citrate) deficiency and the existence of cavities with low urodynamic efficacy. Dietary aspects linked to urinary pH and to the control of urinary phytate and citrate are discussed above and summarized in Table 2. As with hydroxyapatite calculi, these calculi are occasionally associated with tubular acidosis and require pharmacological treatment.

### Uric acid calculi

The most important risk factor for uric acid crystallization and stone formation is a low urinary pH (below 5.5) rather than high urinary uric acid excretion. By controlling urinary pH, uric acid stone disease can be prevented, this being one of the few urinary tract stones that can be successfully dissolved *in vivo*. Thus, the recommendations for uric acid stones involves liquid ingestion to produce daily urine volumes above 2 L, urine alkalinization using citrate or bicarbonate to maintain pH values between 6.2 and 6.5 [41], and a predominantly vegetarian diet. Maintaining a moderate consumption of animal protein, seafood (see Table 5) and alcohol is also important [42]. Attention to food portion size is also important [42]. However, if the calculus is large or is located in cavities with very low urodynamic efficacy, dissolution via urinary alkalinization is very difficult, if not impossible. Treatment of this disorder requires periodic control of urinary pH to avoid excess alkalinization, which could cause other problems such as hydroxyapatite lithiasis.

**Table 5:** Main purine-rich foods

Purine Rich Animal Foods [46]
• Seafood
• Canned seafood: anchovies, sardines in oil, herrings.
• Fish roe
• Meat
• Organ meat: liver, kidney, sweetbreads
• Meat extracts, consomme, gravies.

Little is known about the precise identity and quantity of individual purines in most foods, especially when they are cooked or processed. In addition, the bioavailability of various purines contained in different foods varies substantially.

### Calcium oxalate/uric acid mixed calculi

The main etiologic factors related to this type of renal calculi are urinary crystallization inhibitor (citrate, phytate) deficiency, urinary pH values below 5.5 and the presence of renal cavities with low urodynamic efficacy. Due to the double effect of citrate acting as a crystallization inhibitor and increasing the urinary pH, citrate-rich foods or citrate drugs are the basis of the most effective dietary or pharmacological treatment for these calculi [12]. In such cases, the urinary pH must be controlled to avoid high values that could induce hydroxyapatite formation.

### Cystine calculi

Hypercystinuria is due to an autosomic recessive genetic disorder that causes increased renal cystine excretion. The hypercystinuria produces recurrent urolithiasis due to the low solubility of cystine at low urinary pH values. The prophylactic measures are based on a high hydric ingestion (at least 4 L of water daily) and urine alkalinization using potassium citrate. Where these measures are not sufficient, it is possible to use complementary pharmacological treatment [43,44]. A low methionine diet has been proposed to treat hypercystinuria. Methionine is an essential amino acid precursor of cysteine and cystine found in protein from both animal (meat, fish and eggs) and vegetable (soya, wheat and coconuts) sources. However, the preventive efficacy of this diet has not been demonstrated.

## Conclusion

Preventive measures for avoiding each type of renal calculus formation involve specific dietary considerations. The main specific dietary guidelines relating to each lithogen factor and type of renal calculus are summarized in Table 2. While there are specific dietary factors to be considered for each calculus type, there is also a general list of dietary measures that can be recommended in order to avoid any renal calculus formation:

• Daily intake of a suitable liquid volume (minimum 2 L water/day)

• Avoid strictly vegetarian diets

• Avoid excessive animal protein diets

• Avoid excessive salt (NaCl) consumption

• Avoid excessive vitamin C and/or vitamin D consumption

• Consume phytate-rich products (natural dietary bran, legumes and beans, whole cereals)

• Avoid exposition to cytotoxic substances (i.e., analgesics abuse, residual pesticides, organic solvents and cytotoxic drugs)
